# Resistance of SARS-CoV-2 Omicron BA.1 and BA.2 Variants to Vaccine-Elicited Sera and Therapeutic Monoclonal Antibodies

**DOI:** 10.3390/v14061334

**Published:** 2022-06-18

**Authors:** Hao Zhou, Belinda M. Dcosta, Nathaniel R. Landau, Takuya Tada

**Affiliations:** Department of Microbiology, NYU Grossman School of Medicine, New York, NY 10016, USA; hao.zhou@nyulangone.org (H.Z.); belindamitchell.dcosta@nyulangone.org (B.M.D.); nathaniel.landau@nyulangone.org (N.R.L.)

**Keywords:** SARS-CoV-2 variants, COVID-19, Omicron BA.2, monoclonal antibodies, spike protein

## Abstract

The recent emergence of the Omicron BA.1 and BA.2 variants with heavily mutated spike proteins has posed a challenge to the effectiveness of current vaccines and to monoclonal antibody therapy for severe COVID-19. After two immunizations of individuals with no history of previous SARS-CoV-2 infection with BNT162b2 vaccine, neutralizing titer against BA.1 and BA.2 were 20-fold decreased compared to titers against the parental D614G virus. A third immunization boosted overall neutralizing titers by about 5-fold but titers against BA.1 and BA.2 remained about 10-fold below that of D614G. Both Omicron variants were highly resistant to several of the emergency use authorized therapeutic monoclonal antibodies. The variants were highly resistant to Regeneron REGN10933 and REGN10987 and Lilly LY-CoV555 and LY-CoV016 while Vir-7831 and the mixture of AstraZeneca monoclonal antibodies AZD8895 and AZD1061 were significantly decreased in neutralizing titer. Strikingly, a single monoclonal antibody LY-CoV1404 potently neutralized both Omicron variants.

## 1. Introduction

**Figure 1:**
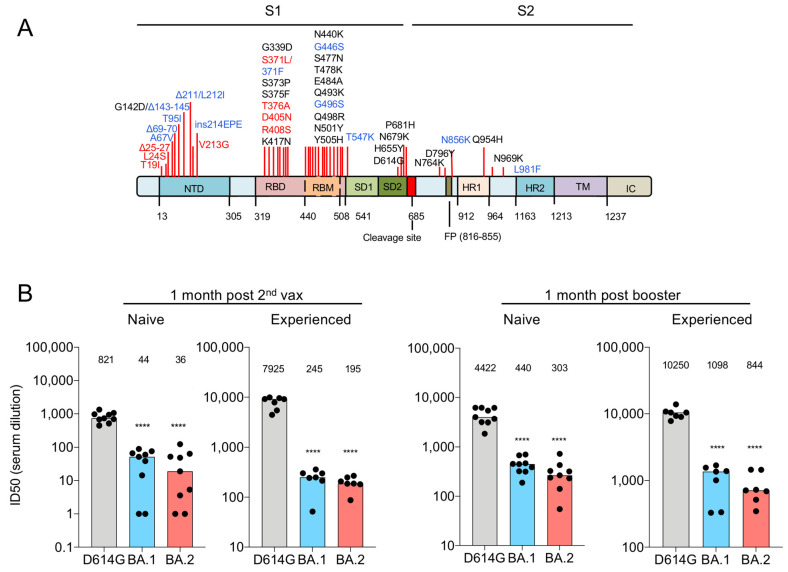
Decreased neutralization of Omicron BA.1 and BA.2 pseudotyped viruses by mRNA vaccine-elicited antibodies. (**A**) The structure of the SARS-CoV-2 Omicron BA.2 spike is indicated. NTD, N-terminal domain; RBD, receptor-binding domain; RBM, receptor-binding motif; SD1 subdomain 1; SD2, subdomain 2; FP, fusion peptide; HR1, heptad repeat 1; HR2, heptad repeat 2; TM, transmembrane region; IC, intracellular domain. Novel mutations found in BA.2 are shown in red. The mutations which are specific to BA.1 are shown in blue. (**B**) D614G, Omicron BA.1 and BA.2 pseudotyped viruses expressing dual GFP/nanoluciferase reporter genes with codon-optimized spike proteins were described previously [19]. Virus were incubated with a 2-fold serial dilution of serum for 30 min and applied to target cells. Luciferase activity was measured two days post-infection. Each serum dilution was measured in duplicates and the experiment was done twice with similar results. Statistical significance was calculated by two-sided testing. (**** *p* ≤ 0.0001). Neutralizing antibody titers of participants without (*n* = 9) or with (*n* = 7) SARS-CoV-2 infection were measured with pseudotyped viruses. Sera were collected from participants 1-month post-second vaccination with Pfizer BNT162b2 1-month post-boost. COVID-19 history was determined by symptoms and a PCR+ test or serology. The sera from SARS-CoV-2 experienced were collected prior to February 2021. Thus, the individuals would have been infected with D614G, Alpha or Iota variant.

The Omicron variant (B.1.1.529) was identified late in 2021 in Denmark, South Africa and India [1] and has since become the predominant variant in most of the world. The highly transmissible variant further mutated to generate sublineages BA.1, BA.2, BA.3, BA.4, and BA.5, with BA.1 and BA.2 responsible for most transmissions worldwide. Although BA.1 and BA.2 are less pathogenic [2,3,4,5,6], BA.1 evades antibody neutralization by the sera of vaccinated donors and escapes neutralization by most of the emergency use authorized therapeutic monoclonal antibodies [7,8,9,10,11,12,13,14,15,16]. BA.2 is 1.5-fold more transmissible than BA.1 and is currently the prevalent variant in the United States (85.9% as of 9 April 2022) [17]. The increased transmissibility of BA.2 is not understood but could be related to immune evasion resulting from its novel mutations. The BA.2 spike protein has most of the mutations in BA.1 plus an additional 4 mutations in the receptor biding domain (RBD) (S371F, T376A, D405N and R408S) and 3 deletions [18] (Figure 1A). Vaccine effectiveness with two doses of ChAdOx1 nCoV-19 and BNT162b2 against the Omicron variant at 25 weeks post vaccination was limited to 0–8.8% [19]. Whereas a booster vaccination with an mRNA vaccine increased effectiveness at 2–4 weeks to 64.4–73.9% which waned at 5–9 weeks to 39.6–64.4% [19]. Few studies have investigated antibody responses to BA.2 and how it evades antibody responses post-infection and vaccination and how it evades monoclonal antibody therapies [7,8,9,20]. 

Of the monoclonal antibodies authorized by the Food and Drug Administration for emergency use [21], Regeneron REGN10933 (Casirivimab) and REGN10987 (Imdevimab), Eli Lilly LY-CoV555 (Bamlanivimab) and LY-CoV016 (Etesevimab), and the GlaxoSmithKline/Vir monoclonal antibody Vir-7831 (Sotrovimab) have been found to be largely inactive against BA.1 and discontinued for clinical use while Evusheld, a cocktail consisting of monoclonal antibodies AZD8895 (Tixagevimab) and AZD1061 (Cilgavimab) formulated for slow release for prophylactic use in immunocompromised individuals [22] retains considerable neutralizing titer against BA.1 [12,22,23]. LY-CoV1404 (Bebtelovimab), identified in a high throughput screen of B-cells from a convalescent individual [23], was given Emergency Use Authorization in February 2022 and is currently used clinically. Those mAbs were licensed for use in the US and have been stopped except for Bebtelovimab. Sotrovimab is still being used in the UK.

In this report, we tested the neutralization of BA.2 by the sera of experienced and naïve vaccinated individuals and by the therapeutic monoclonal antibodies. In addition, we mapped the mutations in BA.2 that account for monoclonal antibody resistance to neutralization. We found that the sera collected from naive individuals 1-month post-second vaccination had 18.7-fold and 22.8-fold decreased neutralizing titers against Omicron BA.1. and BA.2, respectively. A historic infection boosts 5-fold higher neutralizing titer against D614G while a 10–15-fold decrease in titer against Omicron (BA.1 and BA.2) was found when compared to parental D614G. The only therapeutic monoclonal antibody that neutralized BA.2 was LY-CoV1404 which neutralized all variants tested with high titer. The mixture of AZD8895 + AZD1061 retained substantial neutralizing activity against BA.2 but with decreased potency. The findings suggest that LY-CoV1404 will be an effective treatment for COVID-19 in patients infected with BA.2.

## 2. Materials and Methods

### 2.1. Plasmids

pMDL and pRSV.Rev used in the generation of lentiviral pseudotypes have been previously described [24]. To generate pLenti.GFP.NLuc reporter vector, fragment containing a GFP/nanoluciferase cassette separated by a picornavirus P2A self-processing amino acid motif was cloned into the BamH-I and Sal-I sites (Addgene plasmid #17448, provided by Eric Campeau and Paul Kaufman). The SARS-CoV-2 Omicron BA.1 spike expression vector pc.Δ19.Omicron was chemically synthesized in two fragments encoding the codon-optimized open reading frame. The two fragments were amplified with external primers containing a Kpn-I and Xho-I sites. The full-length coding Omicron BA.1 spike sequence was generated by overlap extension PCR. Full-length Omicron BA.1 spike was then digested with Kpn-I and Xho-I and cloned into pcDNA6. The pCMV3-SARS-CoV-2-BA.2-Spike was kindly provided by Sho Iketani and David D. Ho (Columbia University Vegelos College of Physicians and Surgeons) [20]. Spike expression vectors with the individual mutations of the Omicron BA.2 RBD were generated by overlap PCR mutagenesis using the D614G spike expression vector pcCOV2.Δ19.D614G as a template. The DNA sequence of spike expression vectors were confirmed by sanger sequencing.

### 2.2. Human Sera, Monoclonal Antibodies 

BNT162b2-vaccinated sera (naïve) were collected 1 month post-second and boost immunization. Serum samples from previously infected donors (experienced) were collected 1 month post-second and boost immunization with BNT162b2. Participants who reported experiencing COVID symptoms were confirmed as previously infected by direct PCR or anti-N ELISA. The sera from SARS-CoV-2 experienced were collected prior to February, 2021. Thus, the individuals would have been infected with D614G, Alpha or Iota variant. Age and sex of the vaccinated donors are shown in Table S1. Donors participated in clinical studies at the NYU Vaccine Center and sera were obtained with written consent under IRB-approved protocols (18-02035 and 18-02037). Casirivimab, Imdevimab were provided by Regeneron Pharmaceuticals. Bamlanivimab, Etesevimab, Tixagevimab, Cilgavimab, Sotrovimab and Bebtelovimab were obtained from discarded vials.

### 2.3. Cells

HEK293T cells were cultured in Dulbecco’s modified Eagle medium (DMEM) supplemented with 10% fetal bovine serum (FBS) and 1% penicillin/streptomycin (P/S) at 37 °C in 5% CO_2_. ACE2.293T cells are clonal cell-lines that stably express a transfected human ACE2. The cells were maintained in DMEM/1 μg/mL puromycin/10% FBS/1% P/S.

### 2.4. SARS-CoV-2 Spike Proteins Lentiviral Pseudotypes

SARS-CoV-2 spike protein pseudotyped lentivirus stocks were produced by cotransfection of 293T cells with pMDL Gag/Pol vector, pLenti.GFP.NLuc and spike protein expression vectors which deleted 19 amino acid from cytoplasmic tail [24] by using calcium phosphate transfection. One day post transfection, the medium was changed. After 1 day, virus-containing supernatants were harvested and passed through a 0.45 μm filter. The filtered supernatant was concentrated by ultracentrifugation at 30,000× *g* for 90 min at 4 °C. The viruses were resuspended in DMEM containing 10% FBS and normalized for reverse transcriptase (RT) activity [12,24]. 

### 2.5. Neutralization Assay 

For the serum neutralizing assay, 50 μL of serially diluted sera (2-fold diluted in DMEM containing 10% FBS) were incubated with 50 μL of pseudotyped lentiviruses (MOI = 0.2). For the monoclonal antibody neutralizing assay, 50 μL of serially diluted monoclonal antibodies (5-fold diluted in DMEM containing 10% FBS) and incubated with 50 μL of pseudotyped lentiviruses (MOI = 0.2). After 30 min, the mixture (100 μL) was added to ACE2.293T target cells (1 × 10^4^ cells) in a 96 well culture dish. After 2 days, supernatant was removed and luciferase activity was measured with Nano Glo substrate (Promega) and luminescence was read in an Envision 2103 microplate luminometer (PerkinElmer) [12,24]. 

### 2.6. PyMol Analysis

Analyses of the structures of the SARS-CoV-2 spike protein with antibody Fabs was performed with the PyMOL Molecular Graphics System, v2.1.1 (Schrödinger, LLC., Vienna, Austria). The PDB accession codes for the structures shown are 6XDG (REGN10933 and REGN10987), 7KMG (LY-CoV555), 7C01 (LY-CoV016), 7JX3 (Vir-7831), 7L7D (AZD8895), 7L7E (AZD1061), and 7MMO (LY-CoV1404).

### 2.7. Data Analysis

All samples were tested in duplicate. Data were analyzed using GraphPad Prism 8 software. The *p* values are corrected for multiple comparison testing using Welch’s *t*-test. Significance was based on two-sided testing. Confidence intervals are shown as the mean ± SD (* *p* ≤ 0.05, ** *p* ≤ 0.01, *** *p* ≤ 0.001, **** *p* ≤ 0.0001). 

## 3. Results

### 3.1. Viruses with BA.1 and BA.2 Spikes were Partially Resistant to mRNA Vaccine Elicited Antibodies

To evaluate the protective effect of vaccine-elicited antibodies against the Omicron variants, we used spike protein-pseudotyped viruses to measure the neutralizing antibody titers in the sera of individuals without a previous history of SARS-CoV-2 infection (naïve) and those previously infected (experienced) who had been immunized twice followed by a third booster immunization with the Pfizer BNT162b2 mRNA vaccine. Lentiviruses pseudotyped by the D614G, BA.1 or BA.2 and spike proteins were prepared as previously described [24]. The sera of naïve individuals 1-month post-second vaccination had neutralizing titers against BA.1. and BA.2 that were decreased an average 18.7-fold and 22.8-fold compared to the parental D614G, respectively. A booster immunization caused the titer against BA.1 and BA.2 to increase 8.4–10-fold (Figure 1B and Table S1). Neutralizing titers against BA.2 were slightly (1.3–1.5-fold) lower than against BA.1 (Figure 1B and Table S1). Sera from of twice vaccinated experienced participants had neutralizing antibody titers against D614G that were 9.6-fold higher than that of sera from naïve individuals. The titers against Omicron BA.1 and BA.2 were 32–40-fold lower than against D614G but were 5.6-fold and 5.4-fold higher than naive sera. One-month post-boost, serum titers from experienced participants increased for D614G, Omicron BA.1 and BA.2 but remained 9.3-fold and 12.1-fold lower on average than that of D614G virus, respectively. Overall, the results showed that both previous infection and boosting increased neutralizing antibody titers, BA.1 and BA.2 remained about 10-fold resistant as compared to the parental virus. 

### 3.2. BA.1 and BA.2 Were Highly Resistant to Most Therapeutic Monoclonal Antibodies

**Figure 2:**
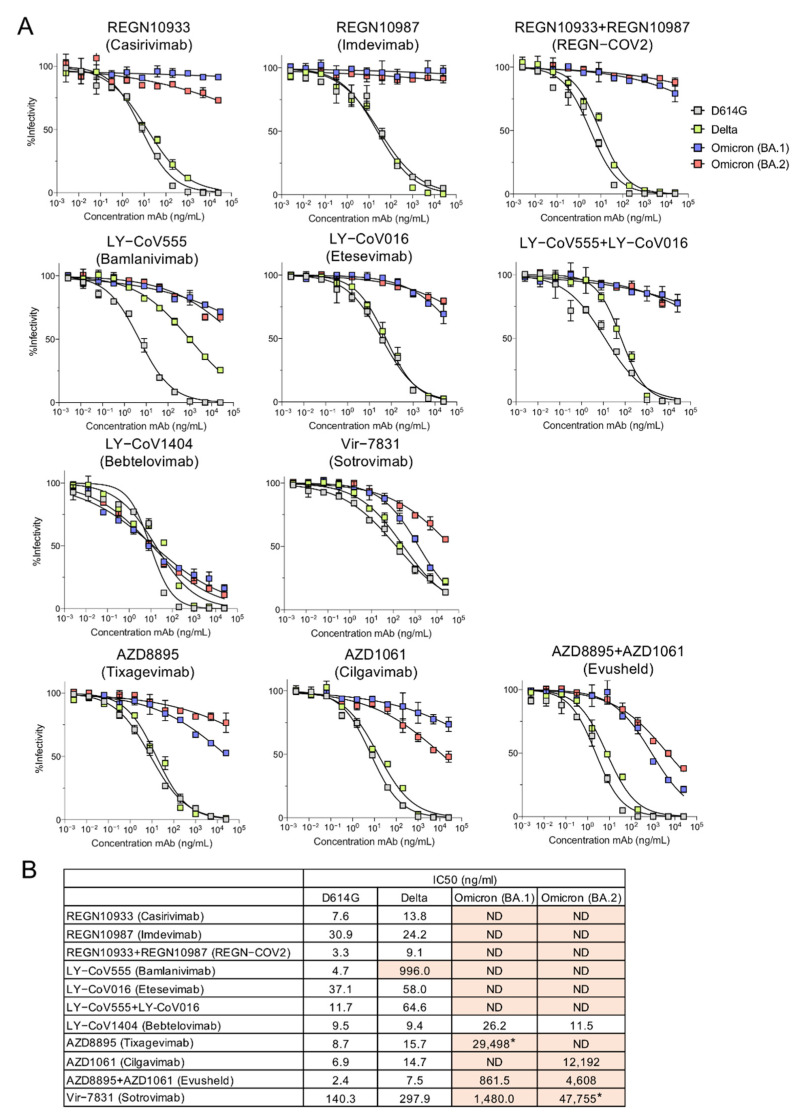
Neutralization of BA.2 by therapeutic monoclonal antibodies. (**A**) Lentiviral pseudotyped viruses were generated as previously described using codon-optimized 19 amino acid deleted spike proteins [24]. A fixed amount of virus, normalized for reverse transcriptase activity, was treated with 5-fold serially diluted monoclonal antibody, in duplicate, for 30 min and then used to infect ACE2.293T cells. Luciferase activity was measured after 24 h and the data are plotted as curves neutralized to luciferase activity in the absence of antibody. (**B**) The IC_50_ was calculated from the neutralization curves by GraphPad Prism 8 software. Values that approached 50% neutralization were estimated, as indicated by an asterisk (*); those that did not approach 50% neutralization were not determined (ND). IC50s with >5-fold decrease in neutralizing titer are shown in orange.

To determine whether the Omicron variants could be neutralized by the therapeutic mAbs, we measured their neutralization with the pseudotyped lentiviruses. Neutralization curves and the calculated IC_50_ values for D614G, Delta, BA.1 and BA.2 pseudotyped viruses are shown (Figure 2A,B). Consistent with previous reports, the mAbs of the REGN SARS-CoV-2 cocktail neutralized D614G and Delta but not BA.1 or BA.2. Similarly, LY-CoV555 and LY-CoV016 neutralized D614G and Delta (although LY-CoV555 had weak activity against Delta) but could not neutralize BA.1 or BA.2. AZD8895 and AZD1061 mAbs which constitute AstraZeneca Evusheld, individually had weak neutralizing activity against BA.1 but in combination, resulting in synergized neutralizing titer, albeit 359-fold lower than against the D614G virus. The antibodies also synergized to neutralize BA.2 although the neutralizing titer was decreased by 1920-fold as compared to the D614G virus. Vir-7831 (Sotrovimab) also showed moderate neutralizing titer against BA.1 but weak neutralizing activity against BA.2 (340-fold decreased compared to D614G, which itself was neutralized with titer 18-fold less than that of REGN10933 on D614G). In striking contrast, LY-CoV1404 potently neutralized all of the variant virus spikes tested. 

### 3.3. Antibody Interaction Sites on the Spike Protein Explain Resistance or Sensitivity to Monoclonal Antibody Neutralization

**Figure 3:**
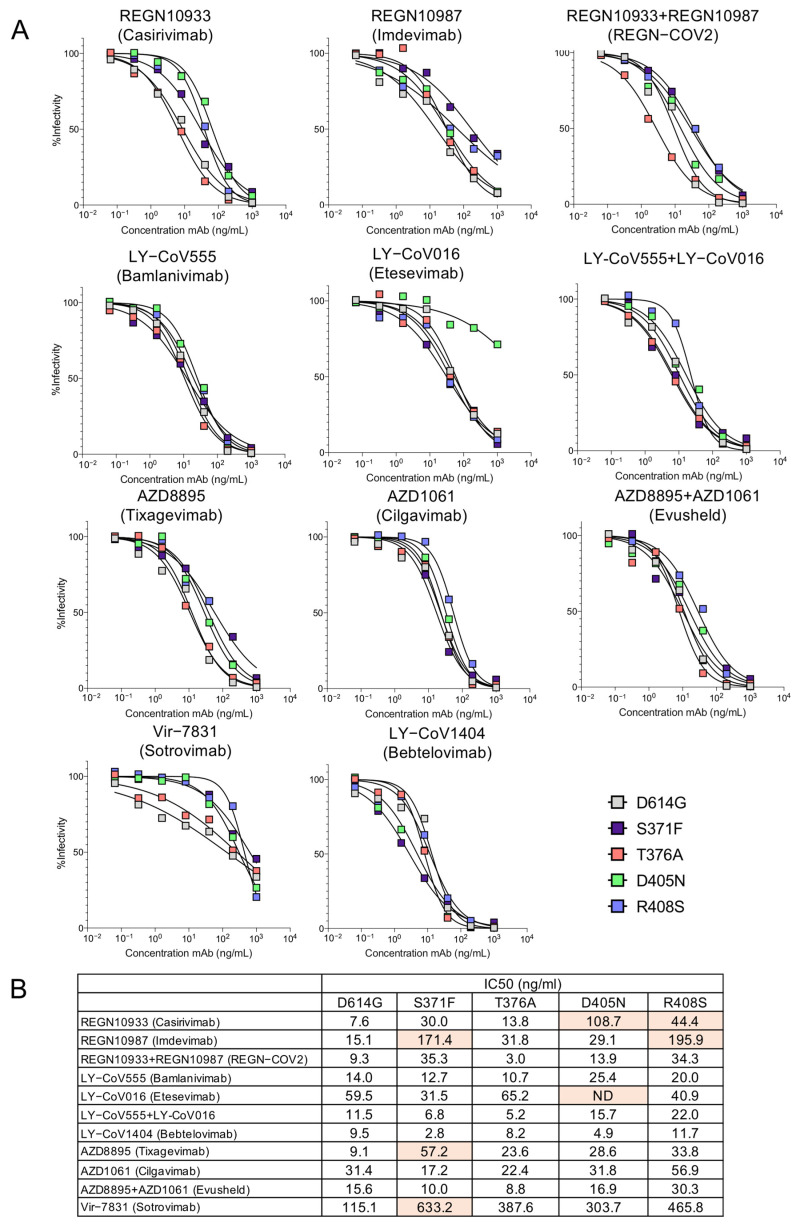
Neutralization of point mutated virus BA.2 by therapeutic monoclonal antibodies. (**A**) Individual point mutated viruses were treated with 5-fold serially diluted monoclonal antibodies and then used to infect ACE2.293T cells. Luciferase activity was measured after 24 h. (**B**) The IC50 was calculated from the neutralization curves using GraphPad Prism 8 software. Mutations found to cause >5-fold decrease in neutralizing titer are shown in orange. Values that did not approach 50% neutralization were not determined (ND).

**Figure 4:**
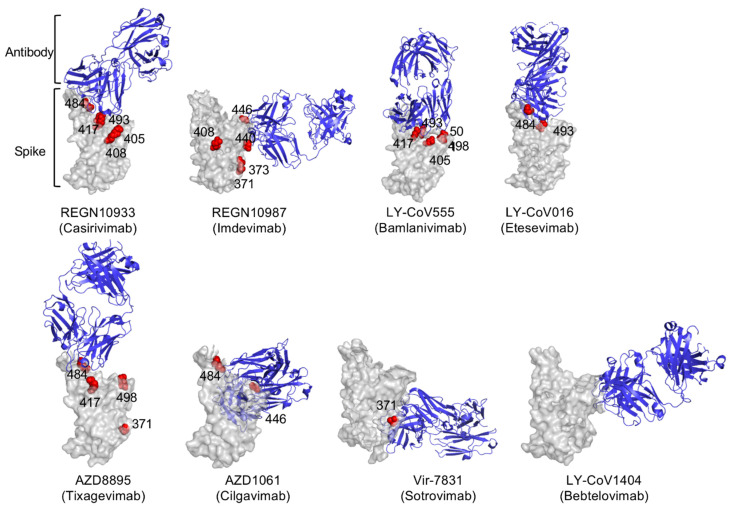
The location of mutations that affect monoclonal antibody binding is shown on the antibody:spike protein complex. Complexes were visualized with PyMOL Molecular Graphics System, v2.1.1 (Schrödinger, LLC) software. Mutations in BA.1 that are also in BA.2 previously reported [12,22,23] to have >5-fold effect on neutralizing titer are shown in red.

To map the mutations in the spike proteins that caused escape from neutralization we tested viruses pseudotyped by spike proteins with the individual BA.2 RBD point mutations (Figure 3A,B). The structural models of the spike protein: antibody complexes are shown with mutations previously found to cause >5-fold decrease in titer against mutations present in both BA.1 [12,22,23] and BA.2. For the most part, the active mutations in BA.1 mapped to the sites of interaction with the antibodies while the additional active mutations in BA.2 (S371F, T376A, D405N and R408S) lie distal to the interaction sites (Figure 4). Presumably, these mutations might affect the conformation of the RBD spike. Interestingly, the LY-CoV1404 epitope does not overlap with the mutations that cause resistance to the other therapeutic antibodies, explaining why the monoclonal antibody retains its ability to neutralize the variants. 

## 4. Discussion

The remarkable and rapid appearance of the Omicron variant has posed considerable challenge both for vaccine development for SARS-CoV-2 and the development of therapeutics to treat COVID-19. Vaccination followed by boosting increases neutralizing titers against the parental and variant viruses, a result which suggests that the booster vaccination will be encouraged to get higher neutralization titer in fully vaccinated individuals or SARS-CoV-2 exposed individuals. The Omicron variants BA.1 an BA.2 were also resistant to nearly all of the monoclonal antibodies tested with the striking exception of LY-CoV1404 which potently neutralized all of the variants tested including BA.1 and BA.2. The ability of LY-CoV1404 to neutralize Omicron is the result of its binding to the spike protein at a site away from the mutated amino acid residues of the variants. The combination of AZD8895 and AZD1061 monoclonal antibodies which constitute Evusheld, a therapy that has been an effective prophylactic that is effective for as long as six months, retained a significant amount of neutralizing activity against BA.1 and BA.2. The effectiveness of the cocktail depends on its concentration in respiratory systems over time. While the monoclonal antibodies individually had low titers, the neutralizing titer of the cocktail against BA.1 and BA.2 resulted from a synergy between the two monoclonal antibodies. 

We previously reported on the mutations in Omicron BA.1 that cause its escape from the monoclonal antibodies [12]. We found that REGN10933 (Casirivamab) was affected by D405N, R408S, K417N, E484A and Q493K mutations. REGN10987 (Imdevimab) was affected by S371F/L, S373P, R408S, N440K, G446S mutations. LY-CoV016 (Etesevimab) was largely affected by D405N, K417N, Q493K, Q498R and N501Y mutations. LY-CoV555 (Bamlanivimab) was slightly affected by E484A and Q493K mutations. AZD8895 (Tixagevimab) was affected by S371F, K417N, E484A and Q498R mutations. AZD1061 was affected by G446S and E484A mutations. The Evusheld cocktail retained moderate neutralizing potency. VIR-7183 (Sotrovimab) was less neutralized by S371F/L mutation. Mutations such as K417N, E484A, and Q493K are located in the interface with the Fab heavy chain and result in escape. The mutations K417N, E484A and Q493K lie outside of the interface but may prevent antibody binding due to the loss of hydrogen bonding with the immunoglobulin heavy chain. The findings presented here demonstrate the difficulty of finding a pan-neutralizing monoclonal antibody against SARS-CoV-2. 

The data generated in this study were derived using lentiviral pseudotypes. We have found that the assay closely coincides with data obtained in live virus assays. In a survey of more than 100 sera from recovered individuals, the pseudotyped assay yielded neutralizing antibody titers that are in close agreement with those determined with the live virus plaque reduction neutralization test (PRNT) [25]. A caveat of this study is the possibility that the pseudotyed assay could be less accurate.

The finding that LY-CoV1404 is able to neutralize all of the variants is encouraging and suggests that the antibody will be valuable for the treatment of severe COVID-19. It remains to be seen whether the antibody remains active against future variants that may emerge. It will be important to identify additional mAbs with binding sites that do not overlap with variant mutations that may occur in the future. 

## Data Availability

The data used in this study are available upon request from the lead contact. Any additional information required to reanalyze the data reported in this paper is available from the lead contact upon request.

## Supplementary Materials

The following supporting information can be downloaded at: https://www.mdpi.com/article/10.3390/v14061334/s1, Table S1: Neutralizing titers of sera from BNT162b2 vaccinated individuals.
