# Supplementary material for: Resistance of SARS-CoV-2 Omicron BA.1 and BA.2 Variants to Vaccine-Elicited Sera and Therapeutic Monoclonal Antibodies
Source: Viruses. 2022 Jun 18;14(6):1334. doi: 10.3390/v14061334 (PMC9228817; doi:10.3390/v14061334)
Supplement: Supplementary file 1 [file viruses-14-01334-s001.zip › viruses-1758258-supplementary.pdf]

Table S1. Neutralizing titers of sera from BNT162b2 vaccinated individuals. (IC50 of BNT162b2 elicited antibodies against viruses with D614G, BA.1 and BA.2 spike proteins. Sera were collected from COVID-19 unexperienced and experienced donors. Age, Sex and Comorbidities are shown.

BNT162b2-COVID-19 unexperienced									
				D614G		BA.1		BA.2	
Donor	Age	Sex	Comorbidities	1 month post vax-2	1 month post booster	1 month post vax-2	1 month post booster	1 month post vax-2	1 month post booster
1	34	M	None	1340	4004	76	453	19	327
2	29	F	None	681	3121	14	321	5	239
3	37	F	Allergy	1063	6162	60	678	64	319
4	62	M	Hypertension, Hyperlipidemia	737	3698	52	450	4	429
5	52	F	Hypertension	696	3175	39	703	125	141
6	34	M	Hypothyroidism	971	6192	1	188	1	55
7	38	F	Asthma, Anemia, Tinea versicolor	937	5419	1	467	1	725
8	38	M	None	449	1854	66	316	52	232
9	52	F	None	517	6175	90	387	49	266
Mean (SD)	41 (9)			821 (282)	4422 (1611)	44 (33)	440 (167)	36 (42)	303 (191)

BNT162b2-COVID-19 experienced									
				D614G		BA.1		BA.2	
Donor	Age	Sex	Comorbidities	1 month post vax-2	1 month post booster	1 month post vax-2	1 month post booster	1 month post vax-2	1 month post booste r
1	45	M	Asthma	5425	10443	250	1695	187	1456
2	37	F	None	9572	8991	301	1389	249	517
3	54	F	Hypertension, Obesity	9880	10453	213	1012	268	729
4	54	M	Cardiovascular disease	4419	7811	243	1566	87	1455
5	25	F	None	9158	10831	52	329	188	691
6	42	F	Diabetes, Herpes simplex	9186	9331	359	1364	182	712
7	43	M	None	7834	13891	300	334	207	346
Mean (SD)	43 (10)			7925 (2168)	10250 (1917)	245 (98)	1098 (565)	195 (58)	844 (439)
